# Novel Mutations in the *MKKS*, *BBS7*, and *ALMS1* Genes in Iranian Children with Clinically Suspected Bardet–Biedl Syndrome

**DOI:** 10.1155/2022/6110775

**Published:** 2022-07-21

**Authors:** Roghayeh Dehghan, Mahdiyeh Behnam, Mansoor Salehi, Roya Kelishadi

**Affiliations:** ^1^Department of Genetics and Molecular Biology, School of Medicine, Isfahan University of Medical Science, Isfahan, Iran; ^2^Cellular, Molecular and Genetics Research Center, Isfahan University of Medical Sciences, Isfahan, Iran; ^3^Pediatric Inherited Diseases Research Center, Research Institute for Primordial Prevention of Non-Communicable Disease, Isfahan University of Medical Sciences, Isfahan, Iran; ^4^Child Growth and Development Research Center, Research Institute for Primordial Prevention of Non-Communicable Disease, Isfahan University of Medical Sciences, Isfahan, Iran; ^5^Department of Pediatrics, Child Growth and Development Research Center, Research Institute for Primordial Prevention of Non-Communicable Disease, Isfahan University of Medical Sciences, Isfahan, Iran

## Abstract

Bardet–Biedl syndrome is a rare autosomal recessive form of syndromic obesity which is characterized by retinal degeneration, obesity, polydactyly, cognitive impairment, and renal and urogenital anomalies. In this study, we used whole-exome sequencing (WES) to investigate the underlying mutations in four Iranian children from consanguineous families with a clinical diagnosis of Bardet–Biedl syndrome (BBS). In three out of four children, we identified one previously reported frameshifting variant in the *BBS12* gene (c.265-266delTT, p.L89fs) and two novel nonsense variants in *MKKS* (c.1196T>G, p.L399X) and *BBS7* genes (c.1636C>T, p.Q546X). In the other child, no mutations were detected in known genes for BBS. However, we identified a novel variant in the *ALMS1* gene (c.10996delC, p.Q3666fs) indicative of Alström syndrome. All variants were interpreted as pathogenic according to American College of Medical Genetics and Genomics (ACMG) guidelines and confirmed through Sanger sequencing. In conclusion, our results not only expand the spectrum of mutations in BBS and *ALMS1* genes but also accentuate the importance of genetic testing for differentiating BBS from Alström syndrome.

## 1. Introduction

Bardet–Biedl syndrome (BBS) is an autosomal recessive (AR) ciliopathy characterized by six primary diagnostic features: retinal degeneration, obesity, polydactyly, cognitive impairment, and renal and urogenital anomalies. In addition, BBS has a wide array of secondary features, among which the typical ones are developmental delay, behavioral abnormalities, speech delay, orthodontic anomalies, brachydactyly/syndactyly, ataxia/poor conditioning, liver disease, craniofacial dysmorphism, Hirschsprung's disease, anosmia, cataract, and nystagmus. The clinical diagnosis is usually prompted by the presence of either four primary features or three primary and two secondary features [1–3]. Thus far, approximately a total of 25 causative genes have been identified for BBS that can explain the molecular causes in about 80% of affected families [3–5]. Most of these genes are related to the formation and/or function of cilia. Therefore, many clinical features of BBS overlap with those of other ciliopathies such as Alström syndrome, Meckel-Gruber syndrome, and Joubert syndrome [6].

Whole-exome sequencing (WES) is a powerful, cost-effective, and time-efficient method for molecular diagnosis of genetic syndromes, particularly those with high genetic heterogeneity, such as BBS. It can potentially help identify novel mutations and therefore broaden human knowledge on the genotypic spectrum of genetic diseases. Additionally, for genetic diseases such as BBS in which the underlying genes have not been fully identified, applying WES can lead to the identification of novel genes [7, 8]. Given all these advantages of the method, we used WES for genetic investigation of four Iranian children with clinically suspected BBS.

## 2. Materials and Methods

Four children with clinically suspected Bardet–Biedl syndrome (cases 1–4), all from consanguineous families, participated in this study approved by the ethics committee of Isfahan University of Medical Science. After obtaining informed consent from their parents, we collected peripheral blood from the probands. Genomic DNA was extracted using the QIAamp DNA Mini Kit (Qiagen, Hilden, Germany) according to the manufacturer's instructions. The DNA samples were then sent to Macrogen (Seoul, South Korea) for library preparation using the SureSelect XT Library Prep Kit (Agilent Technologies, CA, USA), followed by sequencing with the Illumina HiSeq 4000 platform (2x100bp, 100x coverage). The obtained FASTQ raw data files were aligned to the human reference genome GRCh38 using the Burrows–Wheeler Alignment (BWA) tool. Single-nucleotide variants (SNVs) and indels were called and filtered using the Genome Analysis Tool Kit (GATK). We applied GATK hard filtering to remove SNVs with low quality score normalized by depth (QualByDepth < 2), low mapping quality (MQ < 40), strand bias (FisherStrand test > 60), bias in mapping quality (MQRankSum < 12.5), and bias in read position of alternative versus reference allele (ReadPosRankSum < −8). For indels, we removed those with FisherStrand test > 200 and ReadPosRankSum < −20. The variants passing these criteria were annotated using ANNOVAR to obtain gene names, functional consequences (i.e., synonymous/nonsynonymous, frameshift, and stop gain), the potential effects on protein function and conservation scores (SIFT, PolyPhen-2, LRT, MutationTaster, MutationAssessor, GERP++, SiPhy, FATHMM, and CADD), and the frequency in the ExAC database.

**Figure 1:**
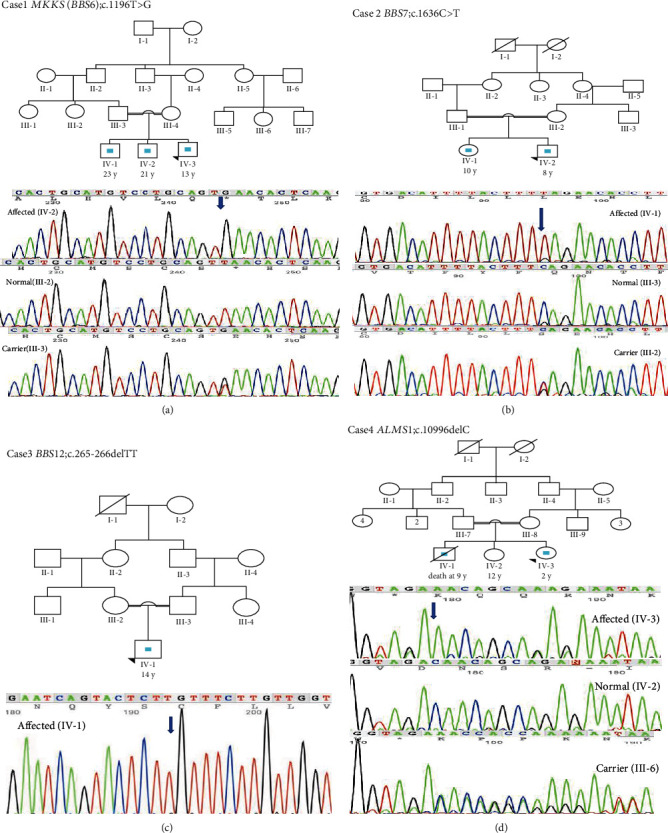
Genetic pedigrees supported the autosomal recessive inheritance of syndromic obesity in the cases. (a, b, d) The Sanger sequencing chromatograms of homozygous affected, homozygous normal, and heterozygous carrier individuals for the novel variants in *MKKS* (*BBS6*), *BBS7*, and *ALMS1* genes. (c) The confirmation of the known pathogenic variant in the *BBS12* gene through Sanger sequencing in the proband.

**Figure 2:**
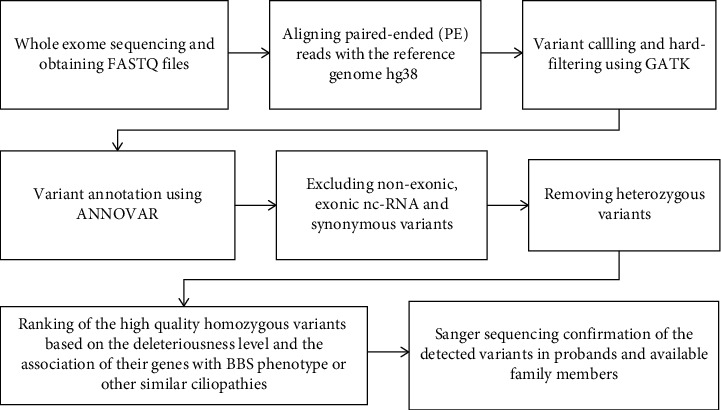
The schematic workflow of WES analysis described from obtaining FASTQ files to Sanger sequencing.

For prioritization, first, we excluded nonexonic, noncoding, and synonymous variants. Then, as the pedigrees supported AR inheritance in all cases, we focused on homozygous variants with high depth of coverage (DP > 20). Finally, we further prioritized variants according to overall ExAC and gnomAD frequency, level of deleteriousness, and the association of their genes with BBS or other similar conditions. Using this approach, we detected one previously reported and two novel loss of function (LOF) homozygous variants (stop gain and frameshifting) in known loci for BBS in three out of four children. In the other child, no deleterious variant was found in known genes for BBS. However, we identified a novel variant in the *ALMS1* gene (c.10996delC) indicative of Alström syndrome. All variants were confirmed through Sanger sequencing in the probands. Additionally, for the novel variants, the available first-degree relatives underwent Sanger sequencing for segregation analysis (Figure 1). All variants were categorized as pathogenic according to American College of Medical Genetics and Genomics (ACMG) guidelines [9] (Table 1). The schematic workflow of WES analysis in our study has been presented in Figure 2.

## 3. Results

The clinical description and genetic testing results of the cases are as follows.

### 3.1. Case 1

The proband was a 13-year-old boy with obesity, bilateral postaxial polydactyly in the hands, progressive retinitis pigmentosa, mild mental retardation (MR), and renal problems. He had two older brothers—one aged 23 and the other aged 20—who had been suffering from the same symptoms but worse retinopathy that had led to blindness. The proband's WES results revealed a homozygous novel nonsense mutation, c.1196T>G (p.L399X), in exon 5 of the *MKKS* (*BBS6*) gene. Sanger sequencing confirmed the homozygous mutation in the proband and his affected siblings and showed that both parents were carriers.

### 3.2. Case 2

This case was an 8-year-old boy presented with obesity, postaxial nubbin in both hands, renal impairment, mild MR, and retinitis pigmentosa. His 10-year-old sister had the same symptoms plus poor conditioning. WES analysis showed that the boy was homozygous for a novel nonsense variant, c.1636C>T (p.Q546X), in exon 7 of the *BBS7* gene. Sanger sequencing confirmed the homozygosity of the affected sister and the heterozygosity of the parents for this variant.

### 3.3. Case 3

This case was a 16-year-old boy with obesity, postaxial polydactyly in the feet, cryptorchidism, distal hypospadias, mild MR, retinitis pigmentosa, and night blindness. He was the only child of a consanguineous marriage. WES analysis revealed that he was homozygous for a previously reported pathogenic variant, c.265-266delTT (p.L89fs), in exon 3 of the *BBS12* gene.

### 3.4. Case 4

This case was a 2-year-old girl with obesity, mild MR, vision problems, and delayed walking who was clinically diagnosed with BBS. She had a normal sister and a deceased brother who had died at the age of 9. Her brother had been suffering from similar symptoms plus hypogonadism. WES analysis in this case revealed a novel homozygous frameshifting variant, c.10996delC (p.Q3666fs), in exon 16 of the *ALMS1* gene. Exon 16 is a mutational hotspot, accounting for 36% of the total mutations in Alström syndrome [10]. Sanger sequencing confirmed the identified variant in the proband and showed that her parents carry this mutation in a heterozygous state.

## 4. Discussion

BBS is a genetically heterogeneous disorder with 25 causative genes identified till date [3]. There is some regional variation in the contribution of each gene to the disease. While mutations in *BBS1* and *BBS10* account for most BBS cases (~23% and ~20%, respectively) in Europe and North America, patients originating from Middle Eastern countries are mostly mutated in *BBS4*, *BBS5*, and *BBS8* [11, 12]. So far, there are only limited reports on the mutation profile of BBS genes in Iranian patients. In the largest study including 14 Iranian families with BBS, related mutations were found in *BBS2*, *BBS4*, *BBS12*, and *BBS9* genes in 28.6%, 14%, 21.4%, and ∼14% of the patients, respectively [13]. In the present report, we used WES for genetic investigation of four Iranian children suspected to have BBS. After genetic testing, we identified two novel and one reported pathogenic variant in the *MKKS*, *BBS7*, and *BBS12* genes in three out of four children. However, in the other child, a 2-year-old girl clinically diagnosed with BBS, we identified a pathogenic variant in the *ALMS1* gene rather than in the BBS genes; therefore, she was diagnosed with another ciliopathy, Alström syndrome.

Alström syndrome is a rare autosomal recessive single gene disorder that shows a great variability in terms of severity and clinical evolution even among patients within the same family. It has a major clinical overlap with BBS and is characterized by cone-rod dystrophy, hearing impairment, childhood obesity, insulin resistance and hyperinsulinemia, short stature in adulthood, cardiomyopathy, and progressive pulmonary, hepatic, and renal dysfunction [14]. Differential diagnosis of Alström syndrome in children could allow clinicians to provide early follow-up examinations for symptoms such as cardiomyopathy and hearing problems that are late-onset presentations of Alström syndrome but are not frequent in BBS [15].

In conclusion, we report three novel homozygous mutations in the *MKKS*, *BBS7*, and *ALMS1* genes in four Iranian children with clinically suspected Bardet–Biedl syndrome. Furthermore, our study accentuates the importance of genetic testing for differentiating BBS from Alström syndrome. The clinical data in addition to genetic testing results of the four cases presented here have not been previously reported. Our results may contribute to the genetic diagnosis and counselling of families with BBS and Alström syndrome.

## Figures and Tables

**Table 1:** The interpretation of variants according to the ACMG guideline. LOF = loss of function.

Case	Candidate variant	The ACMG criteria for pathogenicity met by identified variants			Conclusion
Case 1	c.1196T>G (p.L399X) in *MKKS* (*BBS6*)	Predicted null variant in a gene where LOF is a known mechanism of disease (PVS1)	Cosegregation with disease in multiple affected family members (PP1)	Absent in population databases (PM2)	1 very strong (PVS1) + 1 moderate (PM2) + 1 supporting (PP1) = pathogenic
Case 2	c.1636C>T (p.Q546X) in *BBS7*	Predicted null variant in a gene where LOF is a known mechanism of disease (PVS1)	Cosegregation with disease in multiple affected family members (PP1)	Absent in population databases (PM2)	1 very strong (PVS1) + (moderate (PM2) + 1 supporting (PP1) = pathogenic
Case 3	c.265-266delTT (p.L89fs) in *BBS12*	Predicted null variant in a gene where LOF is a known mechanism of disease (PVS1)	Reported as pathogenic by a reputable source (PP5)	Absent in population databases (PM2)	1 very strong (PVS1) + 1 moderate (PM2) + 1 supporting (PP5) = pathogenic
Case 4	c.10996delC (p.Q3666fs) in *ALMS1*	Predicted null variant in a gene where LOF is a known mechanism of disease (PVS1)	Variant is within a mutational hot spot (PM1)	Absent in population databases (PM2)	1 very strong (PVS1) + 2 moderate (PM1 and PM2) = pathogenic

## Data Availability

The data presented in this study are available on request from the corresponding author.
